# DL-AoD Estimation-Based 5G Positioning Using Directionally Transmitted Synchronization Signals

**DOI:** 10.3390/s25206372

**Published:** 2025-10-15

**Authors:** Ivo Müürsepp, Muhammad Mahtab Alam

**Affiliations:** Thomas Johann Seebeck Department of Electronics, School of Information Technologies, Tallinn University of Technology, 19086 Tallinn, Estonia; muhammad.alam@taltech.ee

**Keywords:** 5G indoor positioning, angle of departure estimation, integrated sensing and communication, IoT localization applications, neural network positioning algorithms

## Abstract

This paper introduces a method for estimating the Downlink Angle of Departure (DL-AoD) of 5G User Equipment (UE) from measured signal strengths of directionally transmitted synchronization signals. Based on estimated DL-AoD values, from two or more anchor nodes, the position of the UE was estimated. Unlike most prior work, which is simulation-based or relies on custom testbeds, this study uses real measurements from an operational 5G network in an industrial factory environment. A deterministic estimator was derived, but multipath and unknown beam characteristics limit its accuracy. To address this, machine learning was applied to automatically adapt to the environment. Previous simulation studies reported 90th-percentile DL-AoD estimation errors below 2°, while experimental works achieved best-case accuracies of 5–6°. In this study, the experimental DL-AoD estimation error remained below 4° for 90% of the measurements, indicating improved real-world performance. Reported positioning errors in the literature range from 3.8 m to 140 m, whereas the 13.2 m error obtained here lies near the midpoint of this range, confirming the practicality of the proposed method in industrial environments. Compared to existing approaches, this work demonstrates high angular accuracy using only sub-6 GHz beams in a realistic industrial scenario without detailed knowledge of antenna beam patterns and channel state. The findings demonstrate that standard 5G signals can provide accurate indoor localization without additional infrastructure, offering a practical path toward cost-effective positioning in industrial IoT and automation.

## 1. Introduction

Fifth-generation (5G) mobile technology offers numerous promising applications, one of which is its built-in capability for accurate mobile positioning. The Third Generation Partnership Project (3GPP) Radio Access Network (RAN) group has introduced a range of measurements specifically intended for positioning purposes in 3GPP Release 16 [1]. Successive 3GPP releases specify the types of positioning-related measurements, their operational ranges, resolutions, and other relevant parameters. These standards also define procedures for the transfer of positioning data and describe general positioning methodologies. A comprehensive overview of the defined measurements and a variety of 5G-based and 5G-assisted positioning techniques is provided in survey [2].

The use of large antenna arrays—comprising tens or even hundreds of elements—enables alternative approaches to mobile positioning. Spatial signal processing techniques, made possible by these arrays, allow for accurate estimation of the direction of arrival (DoA) of 5G signals [3,4]. In practical implementations, established algorithms such as MUSIC, ESPRIT, and their variants are often employed to obtain high-resolution DL-DoA estimates.

Direction estimation can be performed either at the base station (gNB) side—known as Uplink Angle of Arrival (UL-AoA)—or at the user equipment (UE) side—referred to as Downlink Angle of Departure (DL-AoD). However, estimating DL-AoD presents additional challenges, as typical mobile devices do not incorporate antenna arrays, and the orientation of the UE is generally neither fixed nor known. These constraints limit the applicability of conventional angle estimation methods on the UE side. Potential alternative approaches to address this limitation are discussed later in this paper.

With at least two independent angle estimates, triangulation can be employed to determine the position of the UE. Compared to time-based methods, angle-based positioning techniques are less dependent on signal bandwidth and offer greater robustness to synchronization errors.

Due to various limitations, most existing works on 5G positioning remain theoretical. Findings are typically validated through simulations, often conducted under favorable conditions and/or using simplified channel models, as seen in [1,3,5]. When experimental validation is included, it is usually confined to controlled laboratory environments, often relying on custom-built test setups or even unrelated radio equipment [6,7,8]. As a result, there is limited understanding of the actual accuracy of angle estimation and the achievable positioning performance in real-world scenarios.

In contrast, the strength of this paper lies in its use of measurements collected from a commercial 5G network operating in a real industrial environment, using actual network equipment. The main objective of this study is to evaluate the practically achievable accuracy of Downlink Angle of Departure estimation and the resulting positioning performance under realistic conditions, without relying on idealized assumptions or artificial setups.

### 1.1. State of the Art

Although relevant standardization bodies such as 3GPP have provided a comprehensive overview of available positioning methods, the practical implementation of these methods was left to manufacturers and other stakeholders. Paper [9], closely related to the 3GPP technical report [1], addressed the implementation of angle-based positioning within the 5G standard. It provided a general overview of both Uplink Angle of Arrival (UL-AoA) and Downlink Angle of Departure (DL-AoD) approaches. Indoor positioning results were presented based on simulations as proof of concept; however, the simulated area was small, and favorable conditions such as the presence of Line-of-Sight (LOS) paths and dense network infrastructure were assumed. This was also the one out of two papers here that addressed the sub-6 GHz frequency range.

Paper [3] focused on indoor DL-AoD positioning for industrial Internet of Things (IoT) applications under indoor factory scenario with 18 anchor nodes, similarly like also in [10]. The authors of [3] also discussed methods for mitigating the negative impact of Non-Line-of-Sight (NLOS) conditions on positioning accuracy. Like in [9], the results were derived solely from simulations. Furthermore, the proposed algorithm assumed access to low-level physical-layer information, such as the channel matrix, which may not be available in practice.

A comparable assumption was made in the analytical work [4], which proposed a two-step DL-AoD positioning algorithm for millimeter-wave (mmWave) frequencies. While theoretically well-grounded, the method was not validated through experimental measurements.

Work [11] was focused only on improving estimated DL-AoD accuracy in mmWave frequencies; presented simulations were used to show improvement, not actual estimation accuracy.

Angle measurements are not limited to triangulation-based positioning. When combined with distance measurements, the position of the UE can be determined in polar coordinates, analogous to conventional radar systems. This hybrid approach, along with supporting ray-tracing simulation results, was discussed in detail in [12]. Similarly, ref. [13] explored the combination of angle- and time-based measurements to improve positioning robustness. In scenarios where accurate timing measurements are hindered by insufficient LOS paths, the authors proposed the use of both DL-AoD and Round-Trip Time (RTT) for positioning. However, the focus remained on simulations, and no experimental validation was presented. Moreover, the simulations assumed an idealized LOS-only channel, neglecting scattering and multipath effects.

The conference paper [14] investigated UE positioning in the mmWave band using beam strength measurements, assuming that the UE is equipped with an antenna array. The proposed method employed a two-stage Kalman filter to iteratively refine the position estimate. Simulation results, under idealized conditions, involving constant velocity motion along a straight path and LOS propagation, suggested sub-meter accuracy.

Not all prior work on DL-AoD estimation is purely analytical or simulation-based; several studies have also reported experimental results. In [6], an experimental setup operating in the 60 GHz band was described, involving two access points in a small indoor environment under LOS conditions. Two different DL-AoD estimation algorithms were proposed and evaluated under additional assumptions, such as known antenna radiation patterns and a simplified channel model. Importantly, the experiments were not conducted using actual 5G hardware, but rather IEEE 802.11ad (WiGig) transceivers. Similarly, Ref. [7] employed a commercial 60 GHz Wi-Fi system to evaluate an angle-based fingerprinting method in a small office area (2.5 × 3.5 m) with seven stationary target positions. Different antennae beam patterns, rather than beam scanning, were used to enhance fingerprinting performance.

In [15], an Ericsson 5G testbed operating at 15 GHz with 48 available beams was used to collect signal strength measurements for outdoor UE positioning. A machine learning approach was applied to estimate the position, achieving an error of less than 10 m in 80% of the test cases. As the setup employed only a single antenna array, the method itself falls under fingerprinting techniques.

Paper [8] explored Simultaneous Localization and Mapping (SLAM) using a 5G mmWave system operating at 28 GHz. Measurements were conducted in an indoor laboratory environment using self-built hardware. AoD estimation was achieved via beam sweeping on both the gNB and UE sides. The experiments assumed idealized conditions, with smooth, reflective surfaces and empty rooms.

Experimental evaluations using mmWave (57–71 GHz) development kits were presented in [16]. The measurements were conducted over a limited area, and the UE was equipped with an antenna array, which is not representative of typical commercial mobile devices. In [17], directional 5G beam channel state information was used for positioning through a fingerprinting method. The best result—achieving sub-centimeter positioning accuracy (below 0.01 m for 95% of the time)—was obtained using the weighted k-nearest neighbors (WKNN) algorithm. However, the system was based on custom-built hardware and was evaluated only at a small number of fixed locations.

There are some works where 5G NR Synchronization signals were used for positioning, just like in current work. In paper [18] these signals were used for initial coarse estimation of the UE location while other methods are used to refine result. Paper [19] used these signals for indoor positioning along with the Wi-Fi, but they were utilized for fingerprinting, not for angle estimation.

### 1.2. Novelty and Contributions

Next, we summarize the key gaps in existing work and outline how the present study addresses them. Both simulation-based and experimental studies in literature are often conducted under simplified assumptions and geometrically favorable conditions. Notably, experimental setups frequently do not utilize actual 5G hardware; for instance, some works relied on Wi-Fi-based equipment such as IEEE 802.11ad systems [7]. In contrast, the measurements in the present study were collected in a live industrial environment, using signals transmitted by commercial 5G network infrastructure. The only deviation from a fully typical usage scenario was the use of a 5G scanner on the UE side for measurement collection.

Furthermore, many experimental studies were limited in spatial scale. For example, Refs. [7,16] reported results from small indoor areas, whereas the present study utilizes data collected over an area exceeding 20,000 m^2^, with the test trajectory alone covering approximately 350 m^2^. In addition, prior evaluations often assessed positioning performance only at a limited number of fixed locations [7]. In cases where continuous trajectories were used, they were typically simple—such as straight-line paths in [14]. In contrast, the measurement trajectory in this work is continuous, covers a larger area, and exhibits more complex spatial characteristics.

Without exception, all practical DL-AoD experiments reported in the literature were conducted in 5G Frequency Range 2 (FR2), i.e., at frequencies above 6 GHz. While this aligns with anticipated future use of FR2 in advanced 5G deployments, most current commercial networks operate in Frequency Range 1 (FR1), below 6 GHz. To the best of the authors’ knowledge, no prior experimental results have been published on DL-AoD-based positioning in the FR1 band. Given the more favorable propagation characteristics of FR1 and the expected coverage limitations of FR2, there remains significant interest in exploring FR1 for practical mobile positioning applications. The present work is, to the authors’ knowledge, the first experimental study to demonstrate DL-AoD-based positioning in a commercial 5G FR1 deployment.

Several previous works, such as [3,4], assumed that the positioning algorithm has access to low-level physical-layer data—such as per-element antenna signal values, channel state matrices, and antenna steering vectors. While such access may be possible for the manufacturers of 5G hardware, it is generally not available to third-party developers or users, which significantly limits the practical applicability of the proposed methods. In contrast, the positioning method presented in this work relies solely on radio signal strength and quality metrics, which are accessible to third parties and routinely measured by standard user equipment (UE). Therefore, the proposed method does not introduce any additional measurement overhead and can even be executed by a passive listening device, such as the scanner used in our study. The UE does not need to be actively registered in the network to perform the necessary measurements.

Another important advantage of the proposed approach is that it does not rely on any specific channel model. Additionally, unlike methods such as [6], which assume precise knowledge of the base station antenna radiation patterns, our method operates independently of this information.

Our experimental results demonstrate that currently deployed 5G New Radio (NR) signals in the FR1 band can be effectively used for UE direction estimation. The DL-AoD estimation error was found to be less than 0.5° for 50% of the cases and below 4° in 90% of cases. Furthermore, this angular information was successfully applied to positioning, resulting in a mean localization error of approximately 5 m.

In summary, this paper presents the first experimental demonstration of downlink angle-of-departure (DL-AoD) estimation using standard 5G synchronization signals in a real industrial environment. Unlike prior studies that rely on theoretical models, simulations, or non-5G experimental setups, this work is based on measurements collected from an operational 5G network. The proposed method employs a lightweight, data-driven approach that achieves sub-degree angular accuracy with sub-6 GHz beams, significantly improving upon the 5–6° accuracies typically reported in the literature. The approach requires no hardware modifications and operates directly on existing network signals. These advances enable practical and scalable indoor positioning in environments where GNSS is unavailable, supporting industrial automation and other mission-critical IoT applications.

The remainder of this paper is structured as follows. Section 2 introduces the implementation of the DL-AoD estimation method, describes the measurement setup and campaign, and presents the machine learning approach used for angle estimation. Section 3 discusses experimental results and performance evaluation, and Section 4 concludes the paper.

## 2. Materials and Methods

Beamforming is a fundamental capability inherently supported by 5G NR technology. Beam management in 5G networks encompasses several key operations, including beam sweeping, beam measurement, beam detection, beam switching, and beam recovery. Of these, beam sweeping and beam measurement are particularly relevant in the context of mobile positioning.

Beam sweeping is performed at the base station by transmitting directional reference signals through multiple spatial beams, which collectively cover the intended service area. These beams are activated sequentially at predefined time intervals. Concurrently, the UE performs beam measurements and reports the signal strength and quality of each received beam back to the base station on a per-beam basis [20]. In the present study, the deployed 5G Radio Units (RUs) support beam sweeping only in the azimuth plane, without elevation control. Consequently, the terms Downlink Angle of Departure (DL-AoD) and azimuth are used interchangeably throughout this paper.

Beam sweeping is the standard mechanism for estimating DL-AoD at the UE side based on received signal measurements. Specifically, the base station transmits *N_b_* reference signals sequentially into distinct spatial directions *φ_n_*, where each direction is associated with a unique Beam Index (BmIx) value *n*. Simultaneously, the UE measures the Reference Signal Received Power (RSRP) values *P_n_* corresponding to each of these beams. The direction *φ_n_* of the beam with the highest recorded RSRP value is taken as a coarse estimate of the DL-AoD

(1) $$\begin{array}{c} \hat{\varphi }=\varphi_{\underset{n}{\mathrm{arg max}}P_{n}},\;n=0,1,\ldots ,N_{b}-1. \end{array}$$

**Figure 1:**
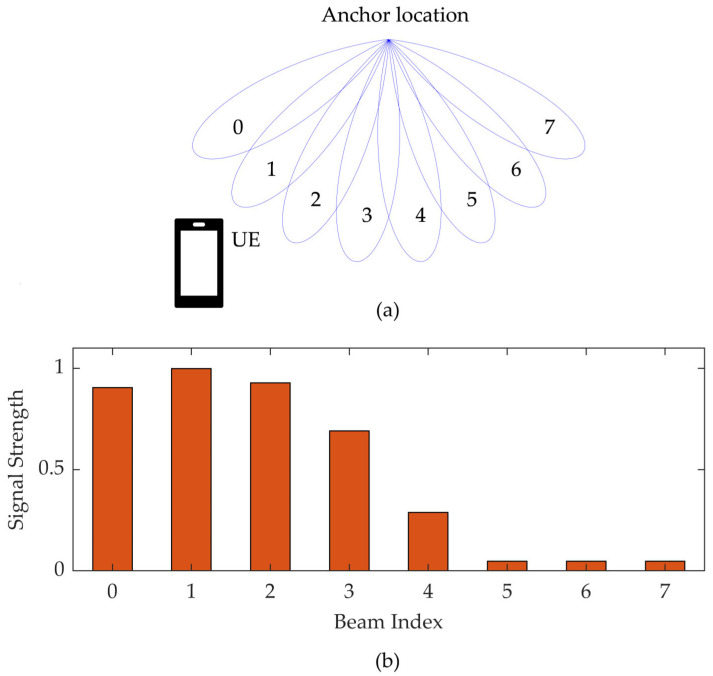
Principle of beam sweeping. Directional beams and location of UE are shown in the upper part (**a**) and corresponding relative signal strengths on the bottom half (**b**).

Figure 1 illustrates the DL-AoD estimation principle using an example scenario in which six directional beams are transmitted within a single sector of a gNB. The corresponding RSRP values measured by the UE are presented as a bar chart at the bottom of the figure. In this example, the highest RSRP value is associated with beam index 1, suggesting that the UE is most likely located in the direction corresponding to that specific beam.

### 2.1. Principle of the DL-AoD Estimation

The angular (azimuth) resolution of the DL-AoD estimation can, in principle, be improved by increasing the number of beams. However, practical constraints—such as the architectural complexity of the transmit antenna array—impose limits on the number of distinct beams that can be supported. Furthermore, transmitting a larger number of beams sequentially increases the overall measurement duration, which in turn reduces the achievable update rate of the positioning system.

To enhance angular estimation accuracy without requiring additional beams or increased measurement overhead, more advanced signal processing techniques can be employed. For example, a closer inspection of Figure 1 reveals that the RSRP values of beams adjacent to the strongest beam (index 1) are nearly equal. This indicates that the UE is located between those beams. A counterclockwise movement of the UE would result in an increase in the signal strength of beam 2 and a corresponding decrease in the signal strength of beam 0. By analyzing the relative signal strengths of multiple adjacent beams, it is possible to interpolate the UE’s azimuthal direction with a resolution higher than the angular separation between beam centers.

For the subsequent analysis and simulations, we adopt a simplified antenna radiation pattern model defined by ITU Recommendation M.2135-1 [21], which is also used in standardized 5G channel modelling frameworks [22]. The relative antenna gain (in dB) is

(2) $$\begin{array}{c} A\left(\varphi \right)=-\min\left[12{\left(\frac{\varphi }{\varphi_{3dB}}\right)}^{2},A_{m}\right]\;, \end{array}$$

where min [.] denotes the minimum function, *φ*_3dB_ is the 3 dB beamwidth and *A_m_* is the maximum attenuation [21].

All signal measurements are conducted at the same physical location, meaning that the path loss component remains constant across all beams. Consequently, variations in the measured signal strength are solely attributable to the directional characteristics of the transmit beam pattern. Let *P*(*φ*) denote the measured signal strength in the azimuth direction *φ* and *A*(*φ*) the relative antenna gain in that direction. Since path loss is identical for all beams and we are concerned only with the relative differences in received power, it is convenient—and possible without loss of generality—to set *P*(*φ*) = *A*(*φ*). For unambiguous interpretation, the analysis is restricted to the angular region where the signal strength exceeds a threshold value *A_m_*. To relate beam-specific measurements to azimuth angle, we incorporate the beam direction *φ_n_* into the formulation

(3) $$\begin{array}{c} P_{n}\left(\varphi \right)=-12{\left(\frac{\varphi -\varphi_{n}}{\varphi_{3dB}}\right)}^{2}. \end{array}$$

**Figure 2:**
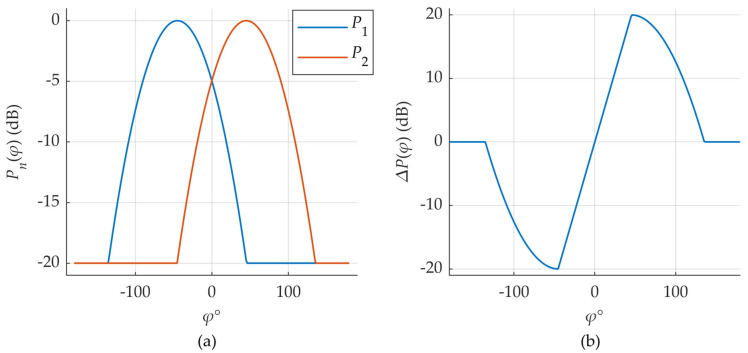
Beam powers (**a**) and their difference (**b**) for an example setup with two beams.

However, the signal strength alone is insufficient for unambiguous azimuth estimation, as it depends on both the distance to the transmitter and the angle of departure. Moreover, because most beam patterns are symmetric around their central direction *φ_n_*, a given signal strength may correspond to two distinct azimuth angles. This ambiguity can be resolved by introducing a second beam oriented in a different direction. An illustrative configuration is shown in Figure 2a, where two beams are centered at *φ*_1_ = −45° and *φ*_2_ = 45°, with a 3 dB beamwidth of *φ*_3dB_ = 70°. While the absolute power levels received from each beam remain dependent on the (unknown) distance to the signal source, the difference in measured power (in dB), denoted Δ*P*(*φ*), is solely a function of the azimuth angle:

(4) $$\begin{array}{c} {\Delta P\left(\varphi \right)=P}_{2}\left(\varphi \right)-\;P_{1}\left(\varphi \right)=\\ \begin{array}{c} =\;-12{\left(\frac{\varphi -\varphi_{2}}{\varphi_{3dB}}\right)}^{2}+\;12{\left(\frac{\varphi -\varphi_{1}}{\varphi_{3dB}}\right)}^{2}\;. \end{array} \end{array}$$

Simplifying the previous equation leads to a more compact form

(5) $$\begin{array}{c} \Delta P\left(\varphi \right)=\frac{12}{\varphi_{3dB}^{2}}\left[2\left(\varphi_{2}-\varphi_{1}\right)\varphi +\varphi_{1}^{2}-\varphi_{2}^{2}\right].\; \end{array}$$

Rearranging the last equation, we can now find the estimate of DL-AoD based on the difference in measured beam powers as

(6) $$\begin{array}{c} \hat{\varphi }=\frac{∆P\varphi_{3dB}^{2}+12\left(\varphi_{2}^{2}-\varphi_{1}^{2}\right)}{24\left(\varphi_{2}-\varphi_{1}\right)}. \end{array}$$

Within the angular region where the beam responses overlap and remain unambiguous, the relationship between the power difference Δ*P* and the angle of departure is approximately linear, as illustrated in Figure 2b. Therefore, estimating the DL-AoD reduces into simply substituting the measured power difference into a linear model. A similar problem, direction-of-arrival estimation based on radio signal strength, is also addressed in [23]. In that work, a cardioid-shaped antenna radiation pattern is assumed, and a quadratic model is derived to estimate the DoA. In contrast, the approach proposed in this paper relies on a linear function, making it conceptually simpler and computationally more efficient.

It is reasonable to assume that beam power measurement errors are normally distributed when expressed in the linear power domain. Consequently, when power is represented in decibels (a logarithmic scale), the corresponding errors follow a log-normal distribution. However, if the mean power is significantly greater than the standard deviation, the log-normal distribution can be closely approximated by a normal distribution.

Assuming that the power measurements of individual beams are normally distributed with equal standard deviation *σ_P_*, the difference between two such measurements, denoted Δ*P*, will also follow a normal distribution, with standard deviation $\sqrt{2}\sigma_{P}$. Since the azimuth estimation Equation (5) is linear with respect to Δ*P*, the standard deviation of the angle estimation error can be directly derived from this relationship as

(7) $$\begin{array}{c} \sigma_{\varphi }=\frac{\sigma_{P}\varphi_{3dB}^{2}}{12\sqrt{2}\left(\varphi_{2}-\varphi_{1}\right)}. \end{array}$$

Given a fixed power measurement accuracy, there are two principal strategies to improve angular estimation accuracy: First, we can use narrower beams. Reducing the 3 dB beamwidth *φ*_3dB_ increases angular resolution. However, to maintain sufficient overlap between adjacent beams, essential for the proposed interpolation method, the angular spacing between beams must also be reduced. This increases estimation accuracy but reduces the effective estimation range. Moreover, because the estimation error is inversely proportional to the angular separation between beams, placing beams closer together partially offsets the benefit gained from narrower beamwidths.

Increasing the angular spacing between neighboring beams can also improve estimation accuracy, but this is again limited by the requirement that adjacent beams must overlap to ensure continuous estimation capability. To avoid gaps in the coverage area the beams must overlap at least half of their beamwidth.

To expand the DL-AoD estimation range while maintaining accuracy, additional directional beams can be introduced. When the number of beams *N_b_* exceeds two, the estimation process proceeds by first identifying the beam with the maximum measured power. Provided this is not the first (*n* = 1) or last (*n* = *N_b_*) beam, it will have two adjacent beams with indices *n* − 1 and *n* + 1. The stronger of these two neighboring beams is then selected, and the estimation Equation (6) is applied using the power difference Δ*P_mn_*, where *m* and *n* denote the selected beam indices. For the initial two-beam case, this reduces to Δ*P*_10_, the difference in measured power between beams 1 and 0.

With enough directional beams, full 360° coverage is theoretically possible, as demonstrated in [23]. In practical mobile positioning scenarios, however, each gNB sector typically covers approximately one-third of the full azimuth range, limiting the DL-AoD estimation span to about 120°. Since the proposed algorithm can estimate the angle only between adjacent beam centers, the outer halves of the first and last beams fall outside the usable estimation region. This reduces the effective azimuth estimation range to approximately 100° per sector in a stand-alone deployment.

Ideally, the Positioning Reference Signal (PRS) would be utilized as the signal of choice for mobile positioning in 5G NR due to its design specifically for such applications. However, in the present study, PRS was not available in the deployed network. As an alternative, the 5G Synchronization Signal (SS) was employed, being the only signal in 5G NR that is always present and periodically transmitted in the downlink direction.

The SS is transmitted in the form of Synchronization Signal Blocks (SSBs), which are organized into burst sets comprising one or more individual SSBs. At least one SSB is always present within the 5G carrier, serving as the primary means for cell detection and initial access. These SSBs are also used to measure beam and cell-level signal power and quality metrics, and they define the coverage area of the 5G NR cell.

In 5G systems equipped with advanced antenna systems supporting beamforming, each SSB within a burst set may be transmitted in a distinct spatial direction within a cell sector. These directional SSBs are uniquely identified by their respective SSB beam indices. The maximum number of SSBs per burst, denoted as *L_max_*, depends on the operating frequency range. For frequencies below 3 GHz the *L_max_* = 4. For 3 GHz < *f* < 6 GHz, *L_max_* = 8 and for FR 2 where *f* > 6 GHz the *L_max_* = 64 [20].

The Reference Signal Received Power based on Synchronization Signals (SS-RSRP) is defined as the linear average of the received power (in watts) across the resource elements carrying the Secondary Synchronization Signal (SSS) [24]. In practice, SS-RSRP values are reported as Layer 1 (L1) measurements, which are available within the range of −140 dBm to −44 dBm with 1 dB resolution [25]. L1 measurements are particularly suitable for procedures requiring fast response times, such as handovers or beam management. These measurements are processed at the physical layer using filtering techniques to reduce noise effects and enhance accuracy.

### 2.2. Indoor Measurement Campaign in Industrial Environment

Measurements were conducted inside the manufacturing hall of a modular wooden house factory, providing a realistic industrial environment. The measurement area was rectangular, approximately 90 m × 220 m in size, with a ceiling height of 11 m. The hall had a single floor and was largely open, with no major internal walls. The building materials primarily consisted of concrete, stone bricks, and sheet metal, contributing to a complex radio propagation environment.

Three rows of concrete columns extended along the hall’s longer dimension, with approximately 25 m spacing between the rows and 6 m between individual columns within each row. The interior layout of the hall was dynamic, frequently changing as house modules were constructed and moved along the production line toward the hall’s exit. Additional sources of movement and obstruction included forklifts, personnel, and telfers operating approximately 7 m above the floor.

The deployed 5G radio network included two Nokia AirScale Osprey Radio Units, both mounted on the same wall of the hall, 78 m apart at an approximate height of 6 m. To enhance the overlapping coverage area, the RUs were tilted 15° inward toward each other. The RUs were connected to a central unit (CU) located in a separate server room via fiber-optic links. The indoor network operated at a carrier frequency of 3434.88 MHz (ARFCN = 628,992). Each RU acted as a 5G base station with a single sector, providing coverage with six directional beams. The approximate beamwidth per sector was 120°. To distinguish between the two sectors, each RU was assigned a unique Physical Cell ID (PCI) value: PCI = 0 and PCI = 3, respectively.

**Figure 3:**
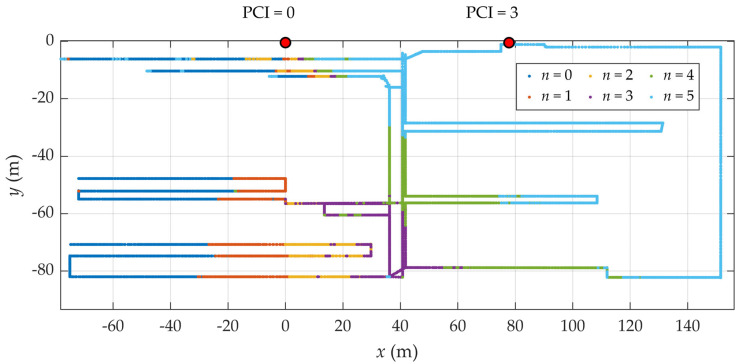
Antenna setup and measurement trajectory inside the industrial test area.

Figure 3 also presents a visualization of the directional beams of the first RU (with PCI = 0) based on the measured SS-RSRP values. At each measurement location, the strongest received beam was identified, and the point was color-coded according to its beam index. For example, a point where beam 0 was strongest appears in dark blue, beam 1 in orange, and so forth. This visualization confirms that a coarse estimate of the user equipment (UE) direction relative to the anchor node can be inferred directly from the index of the strongest measured beam.

However, a more detailed examination reveals that the boundaries between areas associated with different beam indices are irregular and occasionally fragmented. These effects are attributed to shadowing, multipath propagation, and reflections/refractions caused by the complex and dynamic industrial environment.

**Figure 4:**
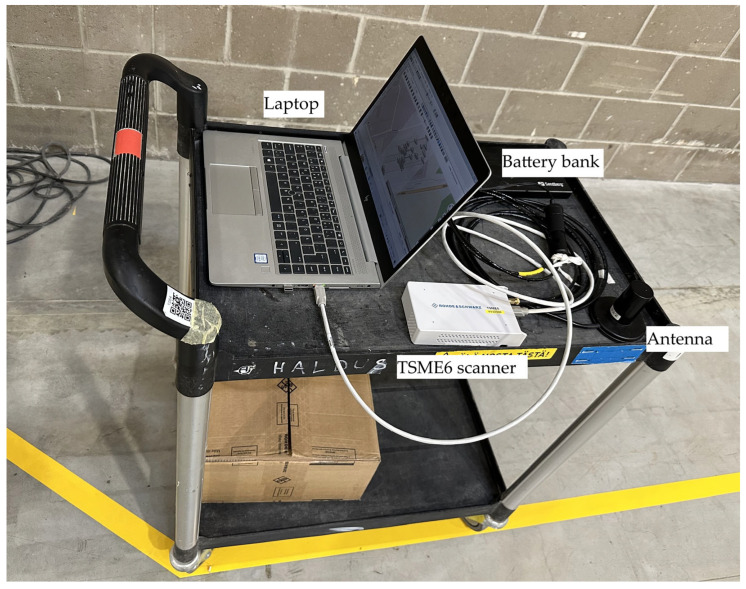
Used indoor measurement setup on a cart. Main components of the used measurement setup are indicated with corresponding labels.

The Rohde & Schwarz TSME6 drive and walk test scanner was used to measure the received strength and quality of the SSB beams. The TSME6 system consists of a main scanner unit and a dedicated 5G antenna. The PCTEL wideband (698–3800 MHz) linearly polarized antenna HA01025-PCRF, visible in Figure 4, was used for later purpose.

As mobile communication systems are designed to operate under random orientations of the user equipment (UE), the effects of signal polarization were not further investigated in this study. Moreover, the polarization of the received signal is typically influenced by reflections and scattering within the propagation environment, making strict polarization alignment impractical in real-world conditions. The base station antennas used in the experiments followed the manufacturer’s standard mounting and polarization configuration. The receive antenna of the measurement scanner was mounted, and therefore polarized, vertically during all measurements.

Data acquisition and analysis were managed via ROMES4 software version 20.0.60, which was executed on a laptop computer connected to the scanner via a Cat6 Ethernet cable. The entire setup was mounted on a hand cart, as illustrated in Figure 4, allowing for convenient mobility throughout the measurement area. The system was powered by LiPo battery banks, ensuring full operational autonomy during the measurement campaign.

In indoor environments, satellite-based positioning systems (e.g., GNSS) are typically unavailable or unreliable due to signal attenuation and multipath propagation.

Therefore, alternative methods must be employed to determine the ground-truth location of the measurement equipment. Several established approaches exist for indoor ground truth acquisition [26].

One such method involves the use of laser distance meters, which are relatively inexpensive and can provide millimeter-level positional accuracy. However, their major limitation lies in the manual nature of the measurements, which makes the process time-consuming and restricts data collection to static points only. An alternative would be the use of LiDAR systems, which enable dynamic localization during movement. Unfortunately, the high cost of such systems rendered them infeasible for the present study.

A more affordable and commonly adopted approach involves the use of floorplans or indoor maps. While this method is less precise than laser measurement, it allows for continuous data collection and has been widely used in practical scenarios. However, it introduces several challenges. The floorplan must be available, geometrically accurate, aligned to a local reference frame, and a reliable method must exist to associate each measurement point with a corresponding location on the map.

In this study, the available factory floorplan was found to be inaccurate and insufficient for localization purposes. Consequently, a new custom indoor map was created. The factory floor contains clearly marked transport routes and pedestrian walkways, delineated by colored lines physically painted on the surface. These markings were used as spatial references for reconstructing an accurate map and for position tagging during the measurement campaign.

An example of such a line, marked on the floor with yellow adhesive tape, is shown in Figure 4. If the coordinates of any two points on a straight line are known, then the entire line segment can be uniquely defined in the local coordinate system. In practice, the positions of the endpoints of each line segment were measured using a tripod-mounted laser distance meter (Leica Geosystems, Switzerland DISTO S910), which enabled highly accurate spatial referencing. More complex trajectories were decomposed into multiple linear sections; each defined in the same manner.

To establish a local reference frame, the location of the first radio unit (RU), with PCI value 0, was selected as the origin. The x-axis was defined to point in the direction of the second RU (PCI = 3), providing a consistent and meaningful orientation for subsequent spatial analysis. Using this reference frame, the ground-truth coordinates of many measurement points along each linear segment could be readily determined by moving the measurement cart along the pre-marked floor lines.

The laser distance meter used for mapping offers a worst-case distance measurement accuracy of ±2 mm. However, the actual accuracy of the resulting ground truth is affected by several additional factors. These include the finite thickness (several centimeters) and minor curvature of the tape lines, manual placement inaccuracies during mapping, and deviations in the cart’s movement, both in terms of velocity and adherence to the line center, during measurements. Taking these factors into account, the total estimated ground truth accuracy is in the order of 1–2 decimeters. Components of estimation error are given in Table A1 in Appendix A.

This level of accuracy is superior to that of standard GNSS receivers in indoor environments and is considered more than sufficient for the objectives of the present study.

The proposed DL-AoD estimation method, as formulated in Equation (6), is expected to perform well in open or idealized environments, if the underlying assumptions are held. In such cases, the estimation accuracy would be primarily limited by the geometrical configuration of the transmit antenna and the measurement accuracy of the beam power. However, in real-world industrial environments, several additional factors introduce complexity and limit the performance of the method.

**Figure 5:**
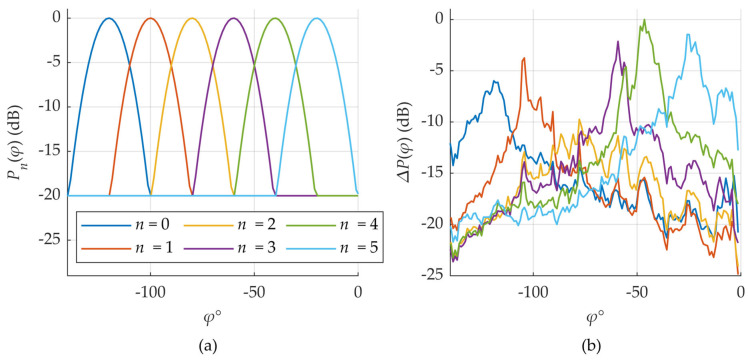
Theoretical beam shapes (**a**) compared against measured (**b**) ones (PCI = 0). Same color for unique beam index *n* is used in both figures.

Specifically, the actual beam shapes are not perfectly known, nor are key parameters such as main lobe direction and beamwidth. Furthermore, multipath propagation, shadowing, blockages, and reflections caused by large metallic or dielectric objects in the environment significantly distort the beam patterns. These effects are visually illustrated in Figure 5.

In the upper plot (a), the theoretical beam patterns for all six beams in a sector are shown, while in the lower plot (b), the actual beam shapes, derived from measured SS-RSRP values, are presented. Despite the environmental distortion, six distinct beam patterns remain clearly identifiable, with maxima located approximately in their expected directions. Nonetheless, the shapes of the beams are highly irregular, and their maximum received signal levels can differ by up to 10 dB, highlighting the influence of environmental effects.

**Figure 6:**
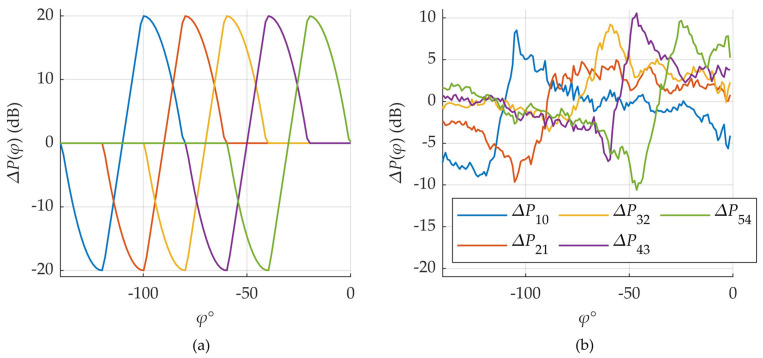
Measured signal strength differences (**b**) compared to the theoretical ones (**a**) (PCI = 0). Same color for unique beam signal strength differences is used in both figures.

A comparison of measured versus theoretical beam power differences is shown in Figure 6. It can be observed that the general trend of the measured curves closely resembles that of the theoretical ones, particularly in the angular region between neighboring beams. This implies that, to a first approximation, the difference in beam powers remains a linear function of the azimuth angle, as predicted by the theoretical model.

These observations support the feasibility of implementing the DL-AoD estimation algorithm based on measured SS-RSRP values. However, to do so, one must first estimate the direction and width of the beams—tasks which are non-trivial in practice due to the previously discussed uncertainties and distortions. The resulting estimation accuracy is presented in the Section 3. Results.

### 2.3. Machine Learning-Based DL-AoD Estimator

While the physical basis of the proposed method is sound, the large number of unknown parameters in real environments motivates the use of data-driven techniques. In such cases, machine learning methods are especially well-suited. In this study, the DL-AoD estimation is framed as a function approximation or function fitting problem, where the mapping from SS-RSRP values to angle of departure is learned from empirical data.

According to the Universal Approximation Theorem, a feedforward neural network (NN) with a single hidden layer can approximate any continuous function over compact subsets of ℝ^n^. Consequently, a one-hidden-layer NN was selected as a baseline for the DL-AoD estimator. To explore potential improvements, two- and three-layer NNs were also tested. Based on evaluation results, the two-layer NN was found to provide the best balance of complexity and performance for the given data.

The activation function for the hidden layers was chosen to be tangent sigmoid (tansig), which is suitable for learning nonlinear transformations. The output layer uses a linear activation function, as the goal is to approximate a continuous-valued output (the DL-AoD) [27].

The input vector to the estimator consists of the measured SS-RSRP values corresponding to the active SSB beams. The target output is the estimated DL-AoD, i.e., the direction of departure corresponding to the signal’s arrival at the measurement location. These values are calculated as

(8) $$\begin{array}{c} \varphi_{i}=\mathrm{atan}\frac{y_{0}-y_{i}}{x_{0}-x_{i}},\; \end{array}$$

where *x_i_* and *y_i_* are the coordinates of *i*-th gNB and *x*_0_, *y*_0_ are actual coordinates of the UE during the measurement. The x-axis of the local indoor coordinate system was selected as the polar axis, corresponding to the zero-azimuth direction.

Both the input features and target values were normalized to the range [−1, 1] prior to use in either training or inference. This normalization range has been shown to yield stable performance, particularly when using the Gaussian–Newton approximation to Bayesian regularization (GNBR) as the training algorithm. The SS-RSRP values were normalized according to their standard reporting range, spanning from −140 dBm to −44 dBm. The azimuth angle, representing the DL-AoD, was normalized over the full circle. The Bayesian regularization, as a robust training method that helps prevent overfitting without the need for a separate validation set, was used. The network weights were initialized using the Widrow–Nguyen method [27], which accelerates convergence for multilayer networks.

The number of input neurons *N_in_* is determined by the number of SSB beams whose signal strengths are used in the estimation. Due to technical limitations of the scanner, the input layer cannot exceed 32 neurons. The output layer consists of a single neuron, corresponding to the estimated DL-AoD.

The number of hidden neurons *N_h_* is not predefined and must be determined experimentally. During training, the GNBR algorithm estimates the effective number of parameters *γ* utilized by the network. If *γ* approaches the total number of weights and biases in the network, this suggests under parameterization, and the value of *N_h_* should be increased. Conversely, if *γ* is significantly smaller than the total number of parameters, the network can be pruned or downsized accordingly, potentially improving generalization.

The training dataset consisted of normalized SS-RSRP values, ordered by beam index within each sector. When data from multiple sectors were included, the sectors were further ordered by increasing PCI value. While measurements were collected across the entire factory floor—including areas outside the angular range of the radio units (RUs), as illustrated in Figure 3—the data were filtered to retain only those measurements that fell within the angular span between the first and last beams of the relevant sector, in accordance with the assumptions of the proposed method. Due to the use of Bayesian regularization, the validation dataset was not needed. For testing purposes a separate dataset, collected over the test trajectory, was used. 

Three different network configurations were evaluated. In simplest case of the single-RU estimation the DL-AoD of each RU was estimated using only the beams originating from that specific RU. Each network had six input neurons, corresponding to the six SSB beams available in a single sector.

In Dual-RU estimation configuration, the estimation was based on the combined beam strengths from both RUs. The resulting networks had twelve input neurons, providing more contextual information to the estimator. This approach leverages the general expectation that data-driven models, such as neural networks, tend to benefit from increased input dimensionality when relevant.

The third setup incorporated beam measurements from an additional, external gNB (PCI = 219), located approximately 800 m from the test site. Although this node was not part of the indoor testbed, its signals were measurable indoors due to leakage. Including this data increased the number of network inputs to sixteen, further enriching the input space and testing the model’s ability to exploit auxiliary information.

**Figure 7:**
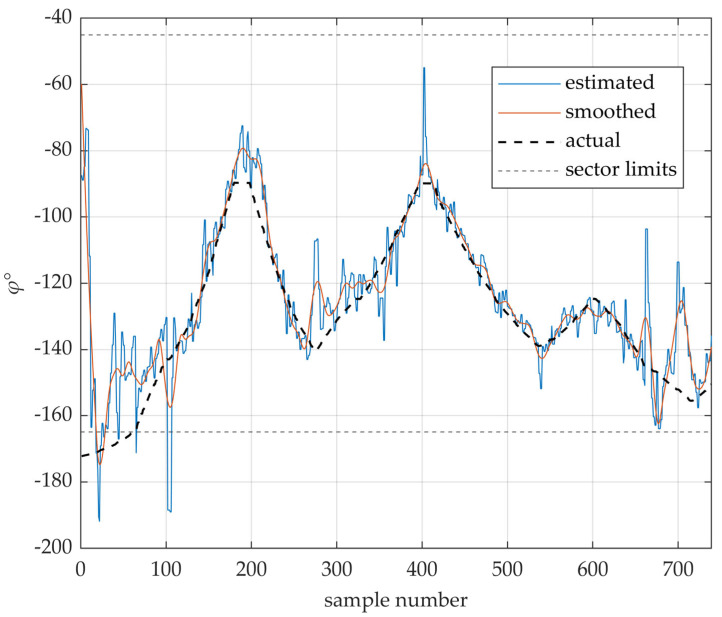
DL-AoD estimation accuracy example (PCI = 3). The actual angular trajectory of the measurement setup is shown with a bold dashed line. Estimated azimuth angles are shown with a blue line, while the smoothed angular trajectory is presented with orange. Limits of anchor node angular coverage are presented with grey dashed lines.

The performance of the proposed DL-AoD estimation method, using the final neural network configuration, is illustrated in Figure 7. The black dashed line represents the ground-truth azimuth angle of the receiver (UE) along the test trajectory, as measured from the second RU (PCI = 3). The blue line shows the corresponding DL-AoD values estimated by the neural network. The thin dashed vertical lines indicate the angular boundaries of the RU’s antenna sector.

As expected, the estimation error becomes significantly large when the UE position falls outside the sector’s angular limits—an inherent limitation of the method, which is designed to operate only within the angular span between the first and last beam. Within this operational range, the estimated DL-AoD closely follows the ground truth, demonstrating the feasibility of the approach. However, some large outliers can still be observed. These may result from local beam distortions, multipath propagation effects, or from inaccuracies in the measured beam strengths.

### 2.4. Triangulation Based on Estimated DL-AoD Values

While the primary focus of this work is on estimating the downlink angle of departure (DL-AoD), it is important to assess how this estimation can be leveraged for user equipment (UE) localization. To demonstrate the practical utility of the proposed method and highlight challenges to be addressed in future work, positioning accuracy was evaluated using triangulation, based solely on the estimated azimuth angles.

Each azimuth measurement *φ_i_* obtained from the *i*th anchor node located at the coordinates (*x_i_*, *y_i_*), defines a straight line with slope *k_i_* = tan(*φ_i_*). When more than one anchor is available, the resulting lines define a system of linear equations. In the presence of measurement errors, this system becomes overdetermined and has no exact solution. However, an estimate for the UE’s coordinates **x** = [*x y*]^T^ can be obtained in the least-squares sense using the standard solution:

(9) $$\begin{array}{c} \hat{x}={\left(A^{T}A\right)}^{-1}A^{T}b, \end{array}$$

where the vector **b** = [*y*_1_ *− k*_1_*x*_1_ *… y_Na_ − k_Na_x_Na_*]^T^ ∈ ℝ*^Na^* ^× 1^ and the matrix **A** ∈ ℝ*^Na^* ^× 2^ is defined as

(10) $$\begin{array}{c} A=\left[\begin{array}{c} \begin{array}{cc} {-k}_{1} & 1\\ -k_{2} & 1 \end{array}\\ \begin{array}{cc} \ldots & \ldots \\ -k_{N_{a}} & 1 \end{array} \end{array}\right]\;. \end{array}$$

Although the proposed DL-AoD estimation method generally provides good angular accuracy (as shown in Figure 7), the triangulation process is highly sensitive to outliers. Since accurate position estimation requires at least two reliable azimuths per point, even a single erroneous angle can significantly degrade the localization result.

In practice, however, the DL-AoD of a moving UE should vary smoothly over time. Therefore, any sudden change in the estimated angle is likely due to measurement noise or inference error and can be treated as an outlier.

Outliers were identified using the Hampel filter, which classifies as outliers any values deviating more than three scaled median absolute deviations (MADs) from the local median using a sliding window of 10 elements. Identified outliers were then replaced via linear interpolation using adjacent, valid values. The remaining data was further smoothed using quadratic regression. The resulting smoothed angular trajectory is shown as the orange line in Figure 7.

**Figure 8:**
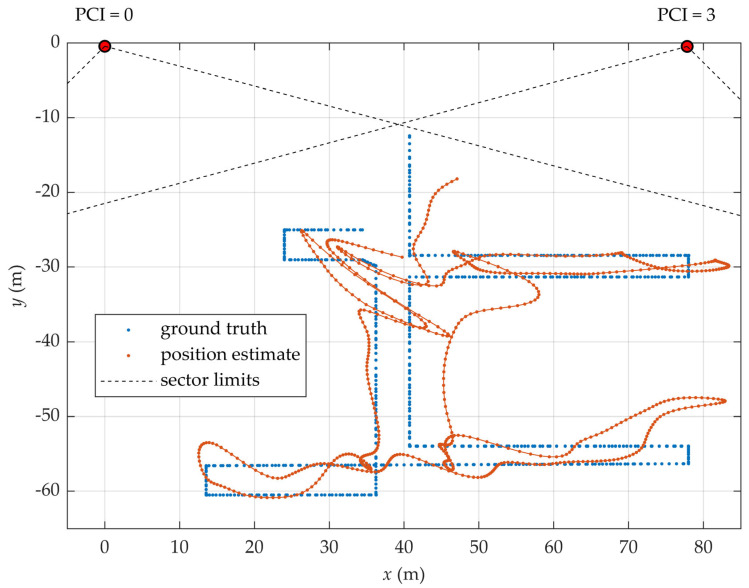
Comparison of triangulation-based position estimate (orange) and ground truth (blue). Measurements from all three anchor nodes (PCI 0, 3, and 219) were used. Test trajectory has 739 measurement points. NN with 16 inputs and two hidden layers (*N*_1_ = 20, *N*_2_ = 50) was used to estimate the DL-AoD.

Positioning accuracy, before and after outlier filtering and smoothing, is summarized at the end of third section. A visual comparison of the estimated test trajectory against the ground truth path is provided in Figure 8.

## 3. Results

The downlink AoD estimation accuracies for different setups are summarized in Table 1 below. For each sector and configuration, the table presents the standard deviation of azimuth estimation errors, along with several percentile values of the error distribution. Specific percentile values were selected in accordance with the ones used in 3GPP TR 38.855 [1], as this simplifies comparison of obtained results with other works. In setups involving machine learning, the neural network configurations are specified in the last two rows of the table, indicating the number of neurons in the first and second hidden layers, denoted *N*_1_ and *N*_2_, respectively.

The first two columns of the table show results obtained using a deterministic algorithm, serving as a baseline for comparison. Although these results are somewhat better than expected given the small number of beams, their performance remains limited. This is primarily due to the test site’s geometric constraints and the absence of accurate information about the actual beam patterns.

The remaining columns of the table present results obtained using neural network-based approaches. Across all setups, estimation errors are effectively zero-mean, and in most cases, 50% of errors are smaller than 0.5°, while 90% remain below 4°. Nevertheless, some large outliers are consistently present, contributing to the relatively high values of the error standard deviation *σ_φ_*.

Table 1 also reveals a clear trend: increasing the number of neural network inputs, by incorporating signal strengths from nearby gNBs, leads to a more compact error distribution. Specifically, lower percentiles of error tend to increase slightly, while the 90th percentile and standard deviation decrease, indicating improved robustness against large deviations. Moreover, using signals from multiple sectors to estimate each DL-AoD value causes the error statistics of both sectors to converge. That is, while the estimation accuracy for anchor PCI = 0 improves, the accuracy for PCI = 3 decreases slightly, leading to more consistent performance between the two.

Compared to the deterministic approach, the azimuth estimation accuracy achieved using neural networks is up to an order of magnitude better, clearly justifying the implementation of machine learning. It is worth emphasizing that the angular separation between the beams is approximately 20°, while DL-AoD estimation errors remain 10 times smaller for 80% of the measurements—demonstrating the model’s ability to interpolate with high accuracy.

The significantly better performance observed for the outdoor base station (PCI = 219) deserves particular attention. The width of the indoor test area is roughly ten times smaller than the distance to this outdoor gNB. As a result, a given angular span corresponds to more training data points in this case, which may contribute to the observed improvement in estimation accuracy. However, since the final positioning accuracy is inversely proportional to the anchor distance, this angular precision advantage is ultimately offset by the geometric disadvantage.

The corresponding positioning accuracies are presented in Table 2. The first row shows the results obtained using raw DL-AoD estimates for triangulation, while the second row presents improvements resulting from outlier removal and trajectory smoothing. The trajectory that is shown in Figure 8 is also based on smoothed DL-AoD values. In some areas the estimated positions closely follow the actual position of the UE, while in others the difference can be quite large. Applied smoothing makes also the estimated trajectory smooth and round-edged. Possible causes and ways of improvement are discussed in next section.

## 4. Discussion

The absence of dedicated reference signals and hardware has thus far hindered the widespread adoption of 5G-based mobile positioning. In this work, we have demonstrated that directionally broadcasted SSB signals, available in standard 5G networks, can be effectively used to estimate the downlink angle of departure. Despite the relatively low carrier frequencies and limited number of beams used in the FR1 band, the resulting estimation accuracy is promising.

The proposed DL-AoD-based positioning method has strong application potential in industrial and IoT settings where GNSS signals are typically unavailable or unreliable indoors. Accurate angle-based localization can support autonomous mobile robots and indoor drones for safe navigation and transport, while also enabling worker safety monitoring, asset tracking, and emergency response. Beyond logistics and safety, precise positioning can enhance collaborative robotics, process optimization, and augmented/mixed reality applications by providing spatial awareness without requiring additional infrastructure. As industrial automation advances, such 5G-based positioning approaches offer a practical, scalable solution for location-aware IoT services.

From a practical standpoint, regular UEs already perform beam measurements and periodically report signal strengths to the network for beam management. Therefore, the proposed approach requires no additional signaling overhead, making it feasible for deployment without modifications to the communication protocol.

When the proposed method is used for other setups, with a different number of anchor nodes or beams available from them, only the number of inputs of NN must be changed and new training data collected from the new location.

The method described here is applicable for estimating azimuth, elevation, or both, depending only on the number and spatial arrangement of available beams. As the FR2 band supports up to 64 beams, even higher estimation accuracy can be expected in mmWave systems. The estimated DL-AoD values can be utilized for pure angle-based positioning via triangulation or integrated with distance measurements, following principles similar to conventional radar systems [12,13].

Although a deterministic expression for DL-AoD estimation was derived, the presence of numerous unknowns in real-world environments makes such methods difficult to apply directly. Machine learning-based approaches, on the other hand, can automatically learn the relevant parameters from training data, leading to substantially improved accuracy. Still, the issue of large outliers remains a significant challenge. While most estimation errors are small, even a single large error can severely degrade positioning performance. Fortunately, since the DL-AoD of a moving object cannot change abruptly, knowledge of UE kinematics can be used to detect and suppress outliers. In this work, we showed that simple filtering and smoothing methods already provide a clear improvement.

### 4.1. Comparison with Existing Works

The results obtained in the present study are next compared with those reported in the literature. As the authors found no experimental studies addressing DL-AoD estimation in the 5G FR1 band, comparisons are made with conceptually similar research.

Starting with angular estimation accuracy, the study in [10] presented simulated results for DoA estimation in a rectangular indoor environment (300 × 150 m) with eighteen anchor nodes. Although the carrier frequency was not specified, the simulation assumes a 100 MHz bandwidth. In that work, 90% of estimation errors were below 2°, with a small number of larger outliers—like the behavior observed in our results.

Moving from simulations to experimental findings, the study in [8] employed eight directional beams of equal angular width within one sector and reported a best-case estimation accuracy of approximately 6°. Likewise, the test setup in [6], which utilized ten beams, achieved around 5° accuracy. In comparison, our results, obtained from real-world measurements in an operational industrial 5G network, demonstrated competitive or superior performance, confirming the feasibility of using existing 5G infrastructure for accurate positioning.

Next, we compare the obtained positioning accuracy against published studies. The results in [3] are presented as cumulative distribution functions (CDFs), from which exact percentiles cannot be extracted; however, the reported horizontal positioning error remains below 0.6 m for 90% of cases. The simulation in [3] assumes a 3.5 GHz carrier frequency and 100 MHz bandwidth, parameters closely matching our setup, and models an indoor factory hall of 120 × 60 × 10 m, comparable to our scenario.

Simulation results reported in [9] cover both FR1 and FR2 frequency ranges using a large indoor open-office model (120 × 50 m). The configuration most similar to our setup involves two base stations acting as anchor nodes, each employing eight directional beams; results for this configuration are summarized in the third row of Table 2. Another simulation study [12] assumes an indoor factory scenario with eight anchor nodes operating in the mmWave range and a 400 MHz bandwidth, achieving a mean-square horizontal positioning error of 2.88 m.

Finally, the technical report [1] compiles findings from multiple industrial contributors regarding NR positioning support, including four separate simulation studies on DL-AoD-based 5G positioning by MediaTek Inc. (Hsinchu, China), Polaris Wireless (Santa Clara, CA, USA), Mitsubishi Electric Co. (Tokyo, Japan), and Qualcomm Inc. (San Diego, CA, USA). MediaTek simulated DL-AoD-based positioning at 4 GHz with a 20 MHz bandwidth and seven anchor nodes under LOS conditions with known gNB beam patterns; results are listed in Table 2. Polaris Wireless conducted a similar study using 4 GHz carrier frequency, eight SSB beams, and a 100 MHz bandwidth, as shown in the fifth row of Table 2. Mitsubishi Electric evaluated angle-based positioning in FR2 (30 GHz) and reported that, when only synchronization signals were used, 90% of positioning errors were below 13 m; incorporating dedicated PRS signals reduced this to 7 m. Interestingly, even under comparable assumptions, reported simulation results differ by up to two orders of magnitude, illustrating the sensitivity of DL-AoD-based positioning accuracy to implementation details and environmental modelling assumptions.

### 4.2. Limitations

Several limitations of the current study should be acknowledged. First, only two indoor radio units (RUs) were available for experimentation. Since at least two anchors are required for triangulation, the RUs had to be positioned apart from each other, which restricted the effective angular coverage to approximately 100°. Although a typical RU sector covers about 120°, the outermost beams could not be fully utilized, as the proposed method requires signals from two neighboring beams. Future work should therefore address this constraint by combining data from multiple sectors to extend angular coverage and enhance robustness.

Second, the number of available directional beams per RU was limited to six, which is fewer than the eight beams supported by the 5G NR standard [20]. Additionally, the placement of anchor nodes was constrained by practical considerations such as access to power supply and data connections, rather than being optimized purely for positioning performance.

Third, ground-truth acquisition methods restricted the measurement area to walkways and transport roads. This limitation was further reinforced by safety requirements, as experiments were conducted in an active manufacturing hall. Finally, both access to network hardware and permission to operate within the factory premises were time-limited, which constrained the total amount of data that could be collected.

The accuracy of triangulation results depends both on the precision of angle estimation and on the positioning geometry, i.e., the relative placement of anchor nodes with respect to the user equipment (UE). The latter effect, commonly referred to as geometric dilution of precision (GDOP), describes how angular estimation errors are amplified by the geometry of the setup. In this study, the test trajectories were chosen in regions with the lowest available GDOP values to minimize this effect.

As noted previously, triangulation results are also highly sensitive to outliers. Even a single erroneous azimuth estimate can lead to a substantial positioning error. Although the overall number of outliers was relatively small, the likelihood of encountering them increases with the number of anchor nodes involved.

Errors in angle estimation may arise from several sources. Some are straightforward, such as measurement noise or limitations in the amount of available training data. More importantly, the factory constitutes a highly dynamic environment: products, raw materials, vehicles, and workers are continuously moving. These changes alter the radio propagation conditions, introducing shadowing, reflections, and scattering effects, which in turn contribute to the observed measurement errors.

### 4.3. Future Work

Although azimuth estimation was the focus of this paper, we also evaluated the resulting positioning accuracy. The performance achieved using raw DL-AoD estimates is modest, but it is important to note that the estimates were entirely independent. Even a basic outlier removal and smoothing strategy significantly enhanced the results. Future improvements could involve applying more advanced filtering techniques such as Kalman filters or particle filters to further refine the trajectory and positioning accuracy. Quantifying sensitivity of localization error to the outlier rate and magnitude is another task ahead.

In the current work, only a single 120° wide sector formed by one radio unit (RU) was utilized. In future studies, the combination of three adjacent sectors of a single gNB will be investigated to achieve continuous 360° angular coverage and improved spatial consistency.

The current study relied solely on SS-RSRP measurements for DL-AoD estimation. Future work should investigate the use of other available metrics, such as signal quality indicators and signal-to-noise ratio (SNR), to improve estimation accuracy. Additionally, the incorporation of quality metrics could enable the estimation of the achievable azimuth accuracy for each anchor node. These estimates could then be used to implement adaptive weighting strategies, improving overall positioning accuracy through geometry-aware localization.

The employed scanner equipment provides a higher dynamic range and resolution than standard commercial UEs. A valuable next step would be to downscale the collected results to UE-grade capabilities to assess their impact on DL-AoD estimation and positioning accuracy. The temporal stationarity of the proposed method remains an open question. Future studies should analyze whether the performance of the DL-AoD estimator degrades over time after training and, if so, characterize the rate of this degradation.

In this work, a shallow neural network was employed as the DL-AoD estimator. Future research should evaluate alternative approaches, including deep learning models, to determine whether they can provide higher accuracy or improved robustness.

## 5. Patents

Patent No. 6200-070BE “Downlink angle of Departure Estimation” was filed in Belgium in 2024 based on the results of the work presented in the current paper.

## Figures and Tables

**Table 1:** DL-AoD estimation accuracies ° summarized.

Setup	Deterministic		ML—Single Sectors		ML—All Indoor Sectors		ML—All Sectors		
PCI	0	3	0	3	0	3	0	3	219
*σ_ϕ_*	9.9	19.9	6.9	11.0	8.0	9.1	6.9	6.3	1.2
50%	5.2	6.2	0.3	0.8	0.4	0.5	0.4	0.4	0.02
67%	7.4	11.5	0.1	1.5	0.8	4.7	0.9	0.9	0.07
80%	10.9	18.6	1.8	3.4	1.9	2.2	2.0	1.9	0.24
90%	14.7	30.4	5.2	7.9	4.1	4.7	4.0	4.2	0.51
*N*_1_	NA	NA	80	40	80	40	80	80	80
*N*_2_	NA	NA	50	50	50	50	50	50	50

**Table 2:** Obtained indoor positioning accuracy (m) and comparison with existing works.

Method	Mean	Std	Max	50%	67%	80%	90%
None	7.3	7.1	43.2	5.2	7.4	9.5	13.2
Smoothed	5.0	3.90	24.7	4.1	5.5	6.9	8.6
[9]				0.5	0.8	1.6	5.0
Ref. [1] Media Tek Inc. (Hsinchu, China)				1.1	1.6	2.0	3.8
Ref. [1] Polaris Wireless (Santa Clara, CA, USA)				59.2	84.3	102.1	140.2

## Data Availability

The raw data supporting the conclusions of this article will be made available by the authors on request.

## Abbreviations

The following abbreviations are used in this manuscript:

3GPP	Third Generation Partnership Project
5G	5th generation of mobile communication systems
BmIx	Beam Index
CDF	Cumulative Distribution Function
CU	Central Unit
DL-AoD	Downlink Angle of Departure
DoA	Direction of Arrival
ESPRIT	Estimation of signal parameters via rotational invariant techniques
FR	Frequency range
gNB	g-Node B–5G base station
GDOP	Geometric Dilution of Precision
GNBR	Gaussian–Newton approximation to Bayesian regularization
GNSS	Global Navigation Satellite System
IEEE	Institute of Electrical and Electronics Engineers
IoT	Internet of Things
ITU	International Telecommunication Union
L1	Layer one—physical layer
LOS	Line Of Sight
MAD	Median Absolute Deviation
MUSIC	Multiple Signal Classification
NLOS	Non-Line of Sight
NN	Neural Network
NR	New Radio—5G radio network standard
PCI	Physical Cell ID
PRS	Positioning Reference Signal
RAN	Radio Access Network
RSRP	Reference Signal Received Power
RTT	Round-Trip Time
RU	Radio Unit
SS	Synchronization Signal
SS-RSRP	Synchronization Signal-based Reference Signal Received Power
SSB	Synchronization Signal Block
SSS	Secondary Synchronization Signal
UE	User Equipment
UL-AoA	Uplink Angle of Arrival

## Appendix A

The endpoint coordinates of each trajectory line marked on the factory floor were measured using a tripod-mounted laser distance meter (Leica DISTO S910) and subsequently stored in a text file. This dataset was later used to generate the ground-truth map, which served both as a reference during the measurement campaign and for post-processing analysis and validation of results.

The local coordinate frame was defined as such that the x-axis aligned with the longest dimension of the manufacturing hall, while the y-axis extended orthogonally along the shorter wall. The origin of the reference frame was assigned to the location of the first radio unit.

The estimated sources of bias in the ground-truth determination, along with their approximate magnitudes, are summarized in Table A1. Assuming that these biases are statistically independent, the combined total bias is estimated to be approximately 12 cm.

**Table A1:** Sources of Ground-Truth Estimation Bias.

Error Source	Estimated Value
Leica DISTO S910 laser distance meter	±0.2 cm
Measurement equipment placement accuracy	±2.5 cm
Deviations of lines on floor	±10 cm
Deviations in cart’s movement	±5.0 cm
Total	±12 cm

## References

[1] (2019). Study on NR Positioning Support.

[2] Mogyorósi F., Revisnyei P., Pašić A., Papp Z., Törös I., Varga P., Pašić A. (2022). Positioning in 5G and 6G Networks—A Survey. Sensors.

[3] Sosnin S., Lomayev A., Khoryaev A. (2021). DL-AOD Positioning Algorithm for Enhanced 5G NR Location Services. Proceedings of the 2021 International Conference on Indoor Positioning and Indoor Navigation (IPIN).

[4] Fascista A., Coluccia A., Wymeersch H., Seco-Granados G. (2020). Low-Complexity Accurate Mmwave Positioning for Single-Antenna Users Based on Angle-of-Departure and Adaptive Beamforming. Proceedings of the ICASSP 2020—2020 IEEE International Conference on Acoustics, Speech and Signal Processing (ICASSP).

[5] Muthineni K., Artemenko A., Vidal J., Nájar M. (2023). A Survey of 5G-Based Positioning for Industry 4.0: State of the Art and Enhanced Techniques. Proceedings of the 2023 Joint European Conference on Networks and Communications & 6G Summit (EuCNC/6G Summit).

[6] Tsai T.-T., Shen L.-H., Chiu C.-J., Feng K.-T. (2020). Beam AoD-Based Indoor Positioning for 60 GHz MmWave System. Proceedings of the 2020 IEEE 92nd Vehicular Technology Conference (VTC2020-Fall).

[7] Koike-Akino T., Wang P., Pajovic M., Sun H., Orlik P.V. (2020). Fingerprinting-Based Indoor Localization with Commercial MMWave WiFi: A Deep Learning Approach. IEEE Access.

[8] Yang J., Wen C.-K., Xu J., Que H., Wei H., Jin S. (2023). Angle-Based SLAM on 5G mmWave Systems: Design, Implementation, and Measurement. IEEE Internet Things J..

[9] Wang Y., Shi Z., Yu Y., Huang S., Chen L. (2019). Enabling Angle-Based Positioning to 3GPP NR Systems. Proceedings of the 2019 16th Workshop on Positioning, Navigation and Communications (WPNC).

[10] Averin I., Bolkhovskaya O., Elokhin A. (2023). Simple Angle-of-Arrive Based Algorithm for Indoor Positioning in 5G Systems. Proceedings of the 2023 Wave Electronics and its Application in Information and Telecommunication Systems (WECONF).

[11] Wang Y., Liu W., Li M., Li C. (2022). A Weighted Iterative Refinement Algorithm for Angle Estimation in 5G Millimeter-Wave Positioning. Proceedings of the 2022 7th International Conference on Multimedia Communication Technologies (ICMCT).

[12] Ahadi M., Kaltenberger F. (2023). 5GNR Indoor Positioning By Joint DL-TDoA and DL-AoD. Proceedings of the 2023 IEEE Wireless Communications and Networking Conference (WCNC).

[13] Guo W., Deng Y., Guo C., Qi S., Wang J. (2022). Performance Improvement of 5G Positioning Utilizing Multi-Antenna Angle Measurements. Satell. Navig..

[14] Rastorgueva-Foi E., Costa M., Koivisto M., Leppanen K., Valkama M. (2018). User Positioning in mmW 5G Networks Using Beam-RSRP Measurements and Kalman Filtering. Proceedings of the 2018 21st International Conference on Information Fusion (FUSION).

[15] Malmström M., Skog I., Razavi S.M., Zhao Y., Gunnarsson F. (2019). 5G Positioning—A Machine Learning Approach. Proceedings of the 2019 16th Workshop on Positioning, Navigation and Communications (WPNC).

[16] Rastorgueva-Foi E., Kaltiokallio O., Ge Y., Turunen M., Talvitie J., Tan B., Furkan Keskin M., Wymeersch H., Valkama M. (2024). Millimeter-Wave Radio SLAM: End-to-End Processing Methods and Experimental Validation. IEEE J. Select. Areas Commun..

[17] Zhou X., Chen L., Ruan Y. (2024). Indoor Positioning with Multibeam CSI from a Single 5G Base Station. IEEE Sens. Lett..

[18] He M., Du K., Huang H., Song Q., Liu X. (2024). BWSAR: A Single-Drone Search-and-Rescue Methodology Leveraging 5G-NR Beam Sweeping Technologies for Victim Localization. Electronics.

[19] Yang H., Chen L., Liu H., Zhu G. (2024). Dynamic Feature-Fused Localization with Smartphones Exploiting 5G NR SSB and Wi-Fi for Indoor Environments. IEEE Trans. Instrum. Meas..

[20] Kottkamp M., Pandey A., Raddino D., Roessler A., Stuhlfauth R. (2022). 5G New Radio: Fundamentals, Procedures, Testing Aspects.

[21] (2009). Guidelines for Evaluation of Radio Interface Technologies for IMT-Advanced. M Series.

[22] (2025). Study on Channel Model for Frequencies from 0.5 to 100 GHz.

[23] Maddio S., Passafiume M., Cidronali A., Manes G. (2015). A Closed-Form Formula for RSSI-Based DoA Estimation with Switched Beam Antennas. Proceedings of the 2015 European Microwave Conference (EuMC).

[24] (2025). NR Physical Layer Measurements.

[25] (2025). Requirements for Support of Radio Resource Management.

[26] Yoshida T., Kaji K., Ogiso S., Ichikari R., Uchiyama H., Kurata T., Kawaguchi N. (2023). A Survey of Ground Truth Measurement Systems for Indoor Positioning. J. Inf. Process..

[27] Hagan M.T., Demuth H.B., Beale M.H., De Jésus O., Martin T. (2014). Neural Network Design.

